# User Testing of the Veteran Delegation Tool: Qualitative Inquiry

**DOI:** 10.2196/40634

**Published:** 2023-02-23

**Authors:** Jolie N Haun, Christine Melillo, Tali Schneider, Marie M Merzier, S Angelina Klanchar, Christopher A Fowler, Rachel C Benzinger

**Affiliations:** 1 James A Haley Veterans Hospital Tampa, FL United States; 2 College of Public Health University of South Florida Tampa, FL United States; 3 Department of Psychiatry and Behavioral Neurosciences Morsani College of Medicine University of South Florida Tampa, FL United States

**Keywords:** electronic health portal, human-centered design, delegate, electronic resources, delegation, care partner, veteran, Veteran Delegation Tool, Veterans Health Administration

## Abstract

**Background:**

Informal caregivers, or care partners, provide critical support to care recipients when managing health care. Veterans Health Administration (VHA) priorities identify care partners as vital in supporting veterans’ care management. The Veteran Delegation Tool (VDT) is VHA’s Health Insurance Portability and Accountability Act–compliant solution for care partners to comanage veterans’ care through VHA’s electronic health portal. Human-centered design approaches in VDT development are needed to inform enhancements aimed at promoting uptake and sustained use.

**Objective:**

The objective of this prospective descriptive quality improvement project was to use a human-centered design approach to examine VDT use perceptions and practical experiences.

**Methods:**

This project was conducted using a 4-phase approach: frame, discover, design, and deliver. The *frame phase* designed the protocol and prepared the VDT system for testing. This paper reports on the *discover phase*, which used semistructured and follow-up interviews and user testing to examine VDT’s benefits, facilitators, and barriers. The *discover phase* data informed the *design and deliver phases*, which are underway.

**Results:**

Veterans (24/54, 44%), care partners (21/54, 39%), and individuals who represented dual roles (9/54, 17%)—namely veteran care partner (4/54, 7%), veteran clinical provider (2/54, 4%), and care partner provider (3/54, 6%)—participated in semistructured interviews in the *discover phase*. A subsample of these participants (3/54, 6%) participated in the follow-up interviews and user testing. Analysis of the semistructured interviews indicated convergence on the respondents’ perceptions of VDT’s benefits, facilitators, and barriers and recommendations for improving VDT. The perceived benefits were authorized access, comanagement of care needs on the web, communication with the clinical team, access to resources, and ease of burden. Perceived barriers were nonrecognition of the benefits of VDT, technical literacy access issues, increased stress in or burden on care partners, and personal health information security. Participant experiences across 4 VDT activity domains were upgrade to My HealtheVet *Premium* account, registration, sign-in, and use. User testing demonstrated users’ challenges to register, navigate, and use VDT. Findings informed VDT development enhancements and recommendations.

**Conclusions:**

Care partners need Health Insurance Portability and Accountability Act–compliant access to electronic health portals to assist with care management. VDT is VHA’s solution, allowing communication among delegates, veterans, and clinical care teams. Users value VDT’s potential use and benefits, while access and navigation improvements to ensure uptake and sustained use are needed. Future efforts need to iteratively evaluate the human-centered phases, *design* and *deliver*, of VDT to target audiences. Continued efforts to understand and respond to care partners’ needs are warranted.

## Introduction

### Background

Care partners can be defined as an “individual, or group of individuals, who assist another person in managing their health care needs on an official or nonofficial basis.” [1] Care partners (eg, spouse, child, and friend) serve a key role in coordinating care for older adults and vulnerable individuals. Emergent priorities within the Veterans Health Administration (VHA) identify this group as a vital resource in veterans’ care management and wellness. However, although an estimated 5.5 million care partners (also known as caregivers) provide crucial support to veterans, they have historically been considered “invisible partners” [2]. Care partners’ involvement facilitates patient-centered care, quality of the care received, and patient satisfaction [3]. Integrating care partners in health care delivery facilitates patient-centered care and improves patient engagement, the quality of care, patient outcomes, and patient satisfaction [4-6].

Care partners often play a critical role helping patients access and manage their health care needs. For example, patients often rely on their care partners to access patient electronic health portals to manage their health care [6,7]. Electronic health portals offer a convenient mechanism for individuals to manage and share health care information, facilitating patient involvement in the process of health care delivery. When granted access to the patient’s electronic health portal, care partners can support care coordination and management of the patient’s health care.

### Access to My HealtheVet

My HealtheVet, the VHA’s electronic health portal for patients, serves over 5.7 million veterans [8]. My HealtheVet offers a variety of web-based tools and resources for veterans to manage their health care and health care–related tasks such as ordering and refilling medications, reviewing laboratory reports and medical records, communicating securely with their health care team, scheduling appointments, and learning about health care topics [9]. Historically, only registered veteran users are permitted to access their My HealtheVet account, but veterans often share the log-in credentials of their My HealtheVet account with their care partners [10,11]. The practice of care partners’ unauthorized access to the veterans’ My HealtheVet account creates both benefit and risk. When granted access to the patient’s portal, care partners can be more involved in care coordination and the management of the patient’s health care [9], but unauthorized access, with no formal safeguards to protect information, presents a potential breach in confidentiality [12].

**Figure 1:**
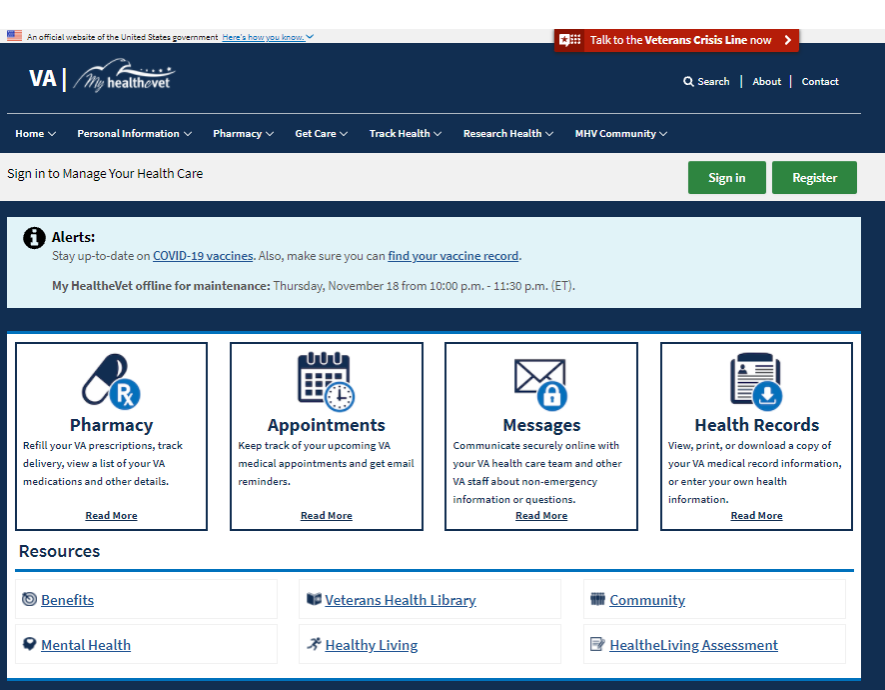
Delegate-permitted functionality. MHV: My HealtheVet; VA: Veterans Affairs.

Recognizing the valuable role care partners play, the Department of Veterans Affairs (VA) is implementing a secure tool with a formalized process designed for veterans to designate trusted individuals (eg, care partners) as delegates to assist in the web-based comanagement of their health care within Health Insurance Portability and Accountability Act regulations, known as the Veteran Delegation Tool (VDT). VDT was designed to allow care partners to comanage essential web-based health care management functions such as managing medications, appointments, communication, and health records to facilitate health care delivery (Figure 1).

The VHA is known for using human-centered design (HCD) to develop, iteratively evaluate, and redesign web-based health resources to inform the development of user-centric tools that are easy to use and useful for the targeted user groups. Our previous work has used HCD and participatory mixed methods to inform the redesign and implementation of VHA’s secured email messaging system (ie, Secure Messaging [13,14]) as well as the redesign of the electronic health portal, My HealtheVet [15-17]. HCD is warranted to understand users’ experiences with VDT to ensure that the tool is useful and easy to use when care partners comanage veterans’ care [18,19]. HCD is an established evidence-based approach to informing product development while prioritizing the experience of the targeted user groups, such as veterans, care partners, and clinical care team members [18]. As part of the implementation of VDT, we collaborated with the VHA operational partners to use an HCD approach to explore users’ perceptions of VDT’s benefits, facilitators, and barriers [19]. The objective of this prospective descriptive quality improvement project was to use an HCD mixed methods approach to examine (1) VDT users’ perceptions about the use of VDT to comanage veterans’ health care and (2) VDT users’ practical experiences using VDT. These data will inform the effective design of a delegation tool that is easy to use and useful for helping care partners comanage care with veterans.

## Methods

This prospective descriptive qualitative study used an HCD framework, with semistructured interviews and user testing, to explore VDT veterans’ perceptions about the benefits of, facilitators of, and barriers to using VDT as well as to examine their practical experiences using VDT. These methods were part of a larger enterprise-wide effort to roll out VDT across the VHA system of care to allow care partners authorized access to My HealtheVet to comanage veterans’ health care.

### Conceptual Framework

The HCD approach puts end users (ie, the people who will ultimately use or benefit from a product or service) at the “center” of the design process by integrating them throughout the process to ensure the uptake and sustained use of the product [20,21]. HCD combines mixed methods to engage users in an iterative design process to ensure the development of high-quality evidence-based tools that are accessible, useful, easy to use, and acceptable to the targeted audiences. In this quality improvement project, the project team collaborated with operational partners to *frame* the issue in preparation for engaging the targeted users in semistructured interviews and VDT user testing. In the *frame phase*, we coordinated with operational partners to prepare the protocol and scripts and prepared the VDT system for user testing.

**Figure 2:**
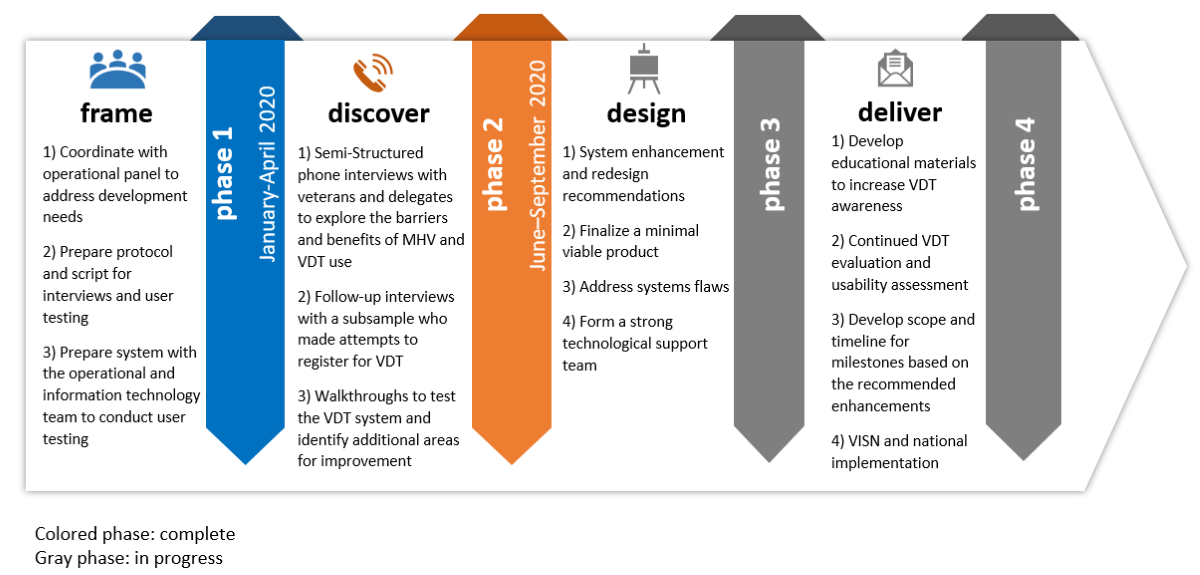
Human-centered design phases. MHV: My HealtheVet; VISN: Veterans Integrated Services Network; VDT: Veteran Delegation Tool.

Next, during the *discover phase*, data were collected for analysis. Emergent themes and recommendations generated through the analysis informed the transition from the *discover phase* to the subsequent *design* and *deliver* *phases,* which are underway. Consistent with HCD, evaluation and progress and milestone assessments are recommended to ensure that the process and outcome measures are met to support the national roll out of VDT across the VHA system of care (Figure 2)*.*

### Setting, Sampling, and Participants

Recruitment for the semistructured interviews was conducted using purposive and snowball sampling at a large Veterans Affairs hospital in the Southeast United States. Veterans and their care partners were identified from the following sources: (1) on-site in services conducted with primary care teams, (2) web-based in services, (3) direct staff referrals, and (4) internal tracking of the report of current registrants. Potential participants were approached via phone. A subsample of the interview participants were asked to participate in the subsequent user testing phase. A subsample of three agreed to participate in user testing. One health care staff became aware of the project and volunteered to be part of the user testing for a staff perspective.

Inclusion criteria included veterans who were aged ≥18 years, who were registered My HealtheVet users, who had no cognitive impairment that prevented the use of a PC or the ability to engage in project activities, and who reported having a caregiver who assisted them with health care management. Inclusion criteria for care partners included those aged ≥18 years, who had no cognitive impairment that prevented the use of a PC or the ability to engage in project activities, and who reported providing caregiving assistance.

On the basis of qualitative sampling methods, saturation was anticipated to occur between 12 and 15 interviews for each VDT user type (ie, veteran and care partner) [22]. An overrecruitment strategy was used to allow for attrition. Up to 25 individuals representing each user type were recruited to ensure saturation across domains.

### Evaluation Team

Our data collection and analysis team is composed of a diverse group of evaluators. TS holds a Doctor of Philosophy degree in educational measurement, evaluation, and research methods. MMM is a registered occupational therapist and holds a Doctor of Philosophy degree. SAK holds a Master of Science degree in nursing education and is a registered nurse. EJB holds a Master of Public Health degree in epidemiology. Keith White holds a medical doctorate. All were employed as health scientists at the time of data collection and analysis. All receive ongoing routine training in qualitative data collection and analyses through their research department and larger system trainings. KW is male. All other team members are female.

Given our recruitment sample, we did not have established relationships with the participants before project commencement. The participants were informed that the purpose of this project was to understand VDT user experiences for process improvement. The information that 2 team members (MMM and SAK) are veterans was shared with the participants and considered while performing analyses.

### Ethical Considerations

The project protocol was reviewed by the Research and Development committee of the James A. Haley Veterans Hospital and deemed *nonresearch* activities to support quality improvement; thus, no informed consent was required. All methods were carried out in accordance with relevant guidelines and regulations. No compensation was provided to the participants in this quality improvement project. The participants’ identities were confidential and deidentified, data were stored behind VA firewalls, and data were presented anonymously in an aggregate form in accordance with the VHA policies and regulations.

### Data Collection Methods and Procedures Within the HCD Framework

#### Data Collection Preparation

In the *frame phase*, the team collaborated with operational information technology system analysts to (1) address development needs, (2) prepare the protocol and scripts for interviews and user testing, and (3) prepare the VDT system for conducting user testing. VDT operation and clinical partner panel sessions were conducted to provide information and educational materials about VDT development and address development needs to prepare for user testing. In tandem, a qualitative methodologist and 5 trained interviewers (3 clinicians and 2 qualitative researchers) practiced and finalized the interview guides (Multimedia Appendix 1). Preparation included activities such as setting up test accounts and system testing to address technical and accessibility concerns identified by the team in preparing the user testing protocol.

In the *discover phase*, a prospective two-step qualitative inquiry was conducted: (1) semistructured interviews and follow-up telephone interviews and (2) user testing using remote audio-visual teleconferencing technology (ie, Microsoft Teams [Microsoft Corp]). In compliance with qualitative sampling standards, participant sampling and data collection continued until saturation was achieved. The procedures followed for data collection have been detailed in the subsequent sections.

#### Semistructured and Follow-up Interviews

A semistructured interview guide was developed to understand the participants’ perceptions about VDT to assess (1) VDT’s facilitators and benefits, (2) VDT’s barriers, and (3) the recommendations to promote and improve VDT. Owing to the timing of the project (from January to September 2020), the impact of the COVID-19 pandemic on the role of care partners and the use of electronic health tools was also addressed. Initial phone interviews lasted approximately 45 to 60 minutes. Field notes were taken, and the interviews were audio recorded for ensuring data quality. During the preliminary interviews, the respondents who expressed willingness to register and participate in VDT user testing were invited to participate in a follow-up interview and user testing.

After the initial interviews, a brief follow-up interview guide was developed to address the topics that emerged during the initial interviews and warranted a follow-up and clarification. A subsample of those who participated in the initial interviews participated in a follow-up telephone interview. The follow-up interviews were approximately 30 minutes long. Finally, the participants of the follow-up interviews were scheduled for a subsequent web-based call to participate in user testing.

#### User Testing

Whereas interviewing the targeted VDT users provided insights into their perceptions about the value of VDT and determinants that will influence their uptake and sustained use of VDT, user testing is an HCD method to practically understand their experiences using the VDT. User testing *walkthrough* sessions were conducted to prompt the participants to complete a series of tasks normally encountered while registering in and using VDT to identify pain points and barriers to accessing, navigating, and effectively completing VDT-related tasks. The tasks comprised the following: (1) upgrade to My HealtheVet *Premium* account, (2) VDT registration, (3) VDT sign-in, and (4) VDT use. User testing was completed on the web using the Microsoft Teams platform [23] to provide live, remote observation with audio and video recording capacity. During the 90-minute walkthrough, the users were asked to complete tasks, including navigating to the VDT site from the invitation email, registering and authenticating for VDT, and sending a message to a designated clinical care provider. The participants were asked to “think aloud” and vocalize their thoughts, experiences, feelings, and opinions while interacting with the tool [24,25]. To capture a complete picture of their experience, screensharing was done and screenshots of the process were collected to demonstrate user flow, ease of use in terms of intuitiveness, and efficiency of the tool. Task completion, obstacles, and facilitators were recorded. After completing the tasks, each respondent discussed their experiences attempting to register in and use VDT to describe challenges and barriers they faced thereof, which helped develop recommendations and strategies to improve the VDT registration process and user flow.

Several design and experiential factors impact user experience; HCD usability factors relevant to content development were used to contextualize domains and subdomains (Textbox 1). As such, Textbox 1 reflects an adapted version of usability factors commonly examined when evaluating websites but was modified for the purposes of evaluating the web-based VDT within the VA’s electronic portal infrastructure [26]. Collectively, these factors can be used to examine user experiences and inform design choices needed to ensure that VDT is easy to use and useful.

**Human-centered design (HCD) usability factors used to contextualize domains and subdomains.**

**HCD usability factors relevant to content development**

- Access: the tool needs to be accessible to all the end users, both veterans and delegates.
- Accuracy: the content needs to be correct and current.
- Affordance: is it evident what the results of a user action will be when the user interacts with an element on your site by clicking, hovering, or tapping it?
- Clarity: the tool needs to provide end users with a clear understanding of the content.
- Ease of comprehension: is the content easy to understand and internalize? Are the sentences and paragraphs concise?
- Intuitiveness: how obvious and easy is the task to accomplish?
- Efficiency: how fast and in how many actions (number of clicks, how much text, etc) can one get to a page of interest?
- Functionality: the tool needs to function appropriately for proper use.
- Learnability: how easy is it for new users to learn the interface and perform a task? For complicated tasks, are there sufficient help features such as tutorials, in-line tips, and hints?
- Utility: the tool needs to provide useful features and functions to the end user.

### Data Management and Analysis

Using Microsoft Excel (Microsoft Corp), data management, coding, and analysis were conducted by 4 team members in a multistep rapid content analysis process [27]. First, a data-coding matrix template was created. The matrix columns followed questions posed in the interview and represented larger domains (eg, benefits and barriers). The interviewers populated the coding matrix with relevant data to incorporate subcodes. Analysts then consolidated the codes to larger categories that shared similar subdomains, drafted the summaries of each category, and provided supporting quotes from the transcript. Data were cross-checked for consistency, accuracy, and reflexivity. To comply with the measures of trustworthiness and rigor of the analysis, the qualitative team met regularly to discuss modifications by adding or removing domain and subdomain categories.

In addition to rapid content analysis, a journey mapping method was chosen to depict veterans’ and delegates’ experiences with the VDT registration process [28]. The journey mapping process identifies various touchpoints in the VDT registration process and interaction with the system that could affect users’ satisfaction and likelihood of use. Given the interaction and reflection required in this final data collection, the participants provided feedback as data collection and understanding evolved. The analysis aimed to identify shared experiences reported by the participants.

## Results

### Demographics

Initial phone interviews were conducted with veterans (24/54, 44%) and their selected care partners (21/54, 39%) in their setting of choice. Notably, some participants (9/54, 17%) represented dual roles, namely veteran care partner (4/54, 7%), clinical care provider veteran (2/54, 4%), and clinical care provider care partner (3/54, 6%). No other people were present beyond the participant and evaluators at the time of data collection. Most veterans were male, whereas most care partners were female. Most participants were older, married, and belonged to nonminority groups. The participant demographics are presented in Table 1. Within each category of Table 1, percentages in each column are based on the participants of each subgroup.

An agreeable subsample (n=3, 2 veterans and 1 care partner) from the initial interview group of 54 participants conducted usability testing with 2 evaluation team members acting as *role players*.

**Table 1:** Demographic information of the Veteran Delegation Tool (VDT) participant sample (N=54).

Characteristics			User roles								
			Veterans (n=24)		Care partners (n=21)		Veteran and care partner^a^ (n=4)		Veteran and clinical provider^a^ (n=2)		Care partner and clinical provider^a^ (n=3)
Age (years), mean (SD)			66.92 (9.59)		63.42 (13.17)		57.00 (18.57)		46.00 (12.73)		59.50 (2.12)
**Sex, n (%)**											
	Female	1 (4)		19 (90)		3 (75)		1 (50)		3 (100)	
	Male	23 (96)		2 (10)		1 (25)		1 (50)		0 (0)	
**Race, n (%)**											
	Asian	0 (0)		1 (5)		0 (0)		0 (0)		0 (0)	
	Black	3 (13)		1 (5)		0 (0)		1 (50)		0 (0)	
	Native American	1 (4)		0 (0)		0 (0)		0 (0)		0 (0)	
	White	20 (83)		13 (62)		4 (100)		1 (50)		3 (100)	
	Missing or declined	0 (0)		6 (29)		0 (0)		0 (0)		0 (0)	
**Education, n (%)**											
	Middle school	0 (0)		1 (5)		0 (0)		0 (0)		0 (0)	
	High school	1 (4)		1 (5)		0 (0)		0 (0)		0 (0)	
	Some college or vocational school	3 (13)		1 (5)		1 (25)		0 (0)		1 (33)	
	Associate degree	1 (4)		2 (10)		1 (25)		1 (50)		1 (33)	
	Bachelor’s degree	3 (13)		6 (29)		0 (0)		1 (50)		1 (33)	
	Graduate degree	3 (13)		1 (5)		0 (0)		0 (0)		0 (0)	
	Missing or declined	13 (54)		9 (43)		2 (50)		0 (0)		0 (0)	
**Employment, n (%)**											
	Disability	4 (17)		0 (0)		0 (0)		0 (0)		0 (0)	
	Full-time employee	0 (0)		3 (14)		0 (0)		2 (100)		1 (33)	
	Unemployed	0 (0)		1 (5)		0 (0)		0 (0)		0 (0)	
	Retired	8 (33)		8 (38)		2 (50)		0 (0)		1 (33)	
	Missing or declined	12 (50)		9 (43)		2 (50)		0 (0)		1 (33)	
**Marital status, n (%)**											
	Married	13 (54)		10 (48)		2 (50)		1 (50)		3 (100)	
	Divorced	3 (13)		1 (5)		2 (50)		1 (50)		0 (0)	
	Single	0 (0)		4 (19)		0 (0)		0 (0)		0 (0)	
	Missing or declined	8 (33)		6 (29)		0 (0)		0 (0)		0 (0)	

^a^A total of 9 participants served dual roles such as veteran and care partner (n=4), veteran and clinical care provider (n=2), and care partner and clinical care provider (n=3).

### Initial and Follow-up Interviews

Data findings were highly convergent among individual user groups, reflecting a shared experience. Likewise, thematic findings across methods, that is, initial and follow-up interviews and user testing, were reflective and convergent. Upon the recruitment of 2 distinct user groups, it was obvious that some participants represented multiple roles. As such, analysis focused on themes within the context of benefits, facilitators, barriers, and the user experience. Ultimately, all data sets were combined and organized into domains and subdomains to inform the examination of VDT’s benefits, facilitators, and barriers within an HCD context. Table 2 illustrates data across themes reflecting veterans’ and care partners’ shared perspectives.

**Table 2:** Exemplar quotes associated with Veteran Delegation Tool’s (VDT) benefits and facilitators.

Subdomain	Veteran quotes	Delegate quotes
Authorized remote access	“...If you couldn’t get to the doctor's office, then we could communicate through the tool.” (Male) “Actually knowing who [the providers] are and being able to provide...what is the appropriate level of information...Updating them...That's huge.” (Male) “I would sign-in and...they know they’re not talking [to the Vet].” (Female)	“I would sign-in and...they know they’re not talking [to the Vet].” (Female)
Comanagement of health care needs on the web	“If I’m hospitalized or I can’t or I don’t remember what prescriptions, he would be able to go in there and figure it out.” (Female)	“There’s a lot of times when he goes to the doctor...he forgets everything...He doesn’t ask any questions from the doctors. If I don’t know something, how am I going to help him?” (Female)
Communication with the clinical team	“[VDT provides] extra communication between the health provider and the physicians and through the VA, and for [care partner] and I to be on the same page. So, if she sees something that I’m not communicating with the physician, she’s able to let them know her concerns about me.” (Male)	“You can write a letter or write a message and send in a request or something needs to be seen or taken care of. It would be easy access instead of calling and leaving a message for the doctor.” (Female)
Resource access and ease of burden	“I would want my daughter to have all of that access, so I think that would be a great benefit for everything. [She is] a lot smarter than me, so when they communicate with her, she’s going to understand better what they’re communicating, and then she’ll tell me in regular English where I can understand it.” (Male)	“It would take some of the stress off of [the Veteran] especially on days that he’s not feeling that well.” (Female)

### Benefits and Facilitators

#### Overview

In total, four subdomains were identified as the benefits and facilitators of VDT use: (1) authorized access, (2) comanagement of health care needs on the web, (3) communication with the clinical team, and (4) access to resources and ease of burden. Collectively, these subdomains describe the benefit of using VDT and the facilitating factors that will support the uptake and sustained use of VDT. Details on these subdomains and relevant transcript excerpts have been presented in the following sections.

#### Authorized Remote Access

All the participants valued the option to expand veterans’ support system by providing care partners formal authorized access to the electronic health portal for comanaging health care while maintaining the protection of personal health information (PHI). Notably, the veterans valued the option to choose the level of access granted to their care partners. In addition to valuing authorized access, the participants reported that VDT was useful given the COVID-19 restrictions. Because many services have become web based and care partners are not allowed during in-person doctor’s visits, VDT is perceived as a valuable resource for comanaging health care, getting clarification when needed, and remaining unexposed to the virus by remotely accessing an authorized secure system.

#### Comanagement of Health Care Needs

Veterans and care partners alike acknowledged the potential for VDT to support comanaging veterans’ health care needs on the web. The participants reported using VDT to facilitate managing medications, schedule and confirm appointments, and review and print laboratory results and medical records. Overall, the participants thought that VDT would facilitate the provision of more efficient care to veterans. The vast majority perceived that VDT could be helpful especially when veterans experience compromised health and are unable to manage their health care independently. VDT was also perceived as helpful in assisting older veterans who are less skilled in using web-based and electronic platforms.

#### Communication With the Clinical Team

Veterans and care partners reported the facilitation of communication as a benefit of VDT, specifically direct communication with the clinical team members. Care partners appreciated the option to communicate with the clinical team to follow-up on clinic visits, request clarifications, and receive updates on health status and medical conditions, especially in cases where veterans and care partners do not reside in the same household or state. Care partners valued the ability to clearly identify themselves when communicating with the care team and facilitate the transmission of appropriate and clear information regarding veterans’ medical care and treatment.

#### Resource Access and Ease of Burden

The respondents reported valuing VDT’s capacity to permit users access to a variety of resources. The ability to independently access veterans’ patient portal and relevant care comanagement resources reduced the perceived burden and stress of both the care partners and veterans. In addition, the care partners valued the ability to express veterans’ concerns on their behalf, specifically for those who are limited in their ability to use the patient portal owing to medical or cognitive reasons.

### Barriers

#### Overview

The participants discussed the potential barriers to using VDT, which reflected four subdomains: (1) nonrecognition of the benefits of VDT, (2) technical literacy access issues, (3) increased stress or burden of care partners, and (4) PHI security (Table 3).

**Table 3:** Exemplar quotes associated with the barriers to Veteran Delegation Tool (VDT).

Subdomain	Veteran quotes	Delegate quotes
Unrecognized benefits	“I don't see the advantages to it [VDT].” (Male)	“I didn’t feel like I needed it. I’m so busy as it is, taking care of them and the house.” (Female)
Technical literacy access issues	“My ability to comprehend...I’m a dinosaur, so it takes us a while.” (Male)	“[some barriers] would be access to the Internet and having devices to be able to do it...and some of the older Veterans and their support system, they’re not tech savvy.” (Female)
Increased stress or burden of care partner	“That’s why she’s kind of forcing me to do all this. She goes, ‘Dad, it just makes everything so much easier. You just got to learn how to do it.’” (Male)	“You’d have to train me on how to use it, how to get in there...It’s just easier to do it on the phone for me, because I can put the phone down, and I can talk...” (Female)
Personal health information security	“I don’t really like [sharing health information through the computer], because I don’t know how secure the computers are.” (Female)	No specific comments

#### Nonrecognition of the Benefits of VDT

A key perceived barrier to potential VDT uptake was a lack of perceived benefits associated with VDT use. Some participants did not recognize the benefit of creating a separate account; for others, telephone communication satisfied their communication needs with providers. Another reason why care partners may not perceive a benefit with using VDT may be the inability to distinguish between VDT and other health care directives. Although the aim of VDT is to provide care partners with authorized electronic access to veterans’ patient portals, many care partners believed that VDT was redundant because of their preexisting health directives. This was mainly true for care partners who were granted release of information, assigned as emergency contact, or had an official power of attorney.

#### Technical Literacy Access Issues

The participants from both groups mentioned difficulties in using web-based systems owing to a lack of understanding and issues with tech literacy. Some participants acknowledged that their lack of technological skills often created challenges. This challenge was exemplified when a veteran referred to himself as a “dinosaur” when using web-based tools. These issues combined with a lack of familiarity with VDT functionality contributed to confusion among user groups. Beyond technology skills, some participants expressed general concerns about accessibility resulting from unreliable internet access or lack of available devices to connect and use the tool.

#### Increased Stress or Burden of Care Partners

A few participants mentioned that using VDT may increase delegates’ burden. A veteran mentioned that their care partner already assists in the management of their general and health-related needs and would not want to overburden their care partner with VDT. In addition, several care partners mentioned that access to medical records and direct communication with clinical providers may be perceived as an intrusion in medical care rather than patient advocacy and can result in potential conflict.

#### PHI Security

Concern with sharing PHI may be one of the only domains predominantly addressed by veterans. This concern was reduced when veterans perceived that they could limit access to certain PHI. In addition to this concern regarding the delegation of access to PHI, veterans expressed a distrust of sharing their medical health data on electronic platforms owing to potential security breaches.

### User Testing: Walkthrough Sessions

#### Overview

During the user testing session, a subsample of users (n=3) discussed their experiences attempting to register in and use VDT. The participants reported their practical experiences during VDT walkthrough sessions across four activity domains: (1) upgrade to My HealtheVet *Premium* account, (2) VDT registration, (3) delegate sign-in, and (4) VDT use. Similar to interview data, user group data sets were highly convergent, as such data sets were combined to illustrate summarized findings. Findings were tabled according to predetermined usability factors; domains, subdomains, identified pain points, and recommendations are presented in Table 4.

**Table 4:** Findings and recommendations.

Domain, subdomain, and pain point				Recommendation
**Domain: upgrade to My HealtheVet*Premium*account**				
	**Utility**			
		Currently nonveteran or non-VA^a^ patient delegates have to select the veteran or VA patient status to be eligible for a My HealtheVet *Premium* account, which is required for delegation	Update options to include all eligible criteria	
	**Accuracy**			
		Site content indicates that nonveteran or non-VA patients are not eligible for delegate registration	Update site language on eligibility criteria	
	**Functionality**			
		The system displays incorrect activity details in the account activity log	Update the system to display correct activity details in the account activity log	
**Domain: VDT^b^ registration**				
	**Access**			
		Delegation invitation only lasts 3 days	Extend invitation to 1 month	
		Lack of a standard operating procedure for in-person registration or for assisting delegates in registering for a sign-in partner	Create a standard operating procedure to assist veterans and delegates	
		Delegation requires My HealtheVet *Premium* account to initiate the registration process	Consolidate the My HealtheVet *Premium* account registration and delegation option	
	**Learnability**			
		Delegation lacks instruction on how to register	Create directions on how to register for delegation	
		Lacks clear indication of steps completed and remaining	Create a progression bar indicating the steps of the registration process	
	**Efficiency**			
		Delegation requires registration for 3 additional sites (ie, Sign-In Partner, My HealtheVet, and My HealtheVet *Premium*)	Streamline the supplemental registration process and provide technological support	
		Delegation authorization process requires multiple signatures	Simplify and streamline the authorization and signature process	
	**Affordance**			
		Delegates do not understand being redirected to My HealtheVet for delegation registration	Provide awareness and technological support for veterans and delegates navigating the registration process	
	**Intuitiveness**			
		Delegation invitation emails contain misleading language	Revise outgoing correspondence to ensure clear language	
	**Functionality**			
		Delegation authorization form processing is not enabled after signature	Enable processing after signature	
		Mismatch of the data requested and systems input data for social security number and date of birth fields	Correct the fields for social security number and date of birth	
		Invitation email not sent right away	Automate invitation email after registration is completed	
**Domain: sign-in**				
	**Intuitiveness**			
		My HealtheVet offers multiple sign-in options without indicating which will provide delegate access	Provide clear instructions on choosing the correct sign-in to view the veteran’s My HealtheVet account	
	**Ease of comprehension**			
		Content on the delegation sign-in page is difficult to understand for registrants and users	Revise the delegation sign-in page content to ensure clarity and comprehension for diverse literacy levels	
	**Functionality**			
		Delegate is unable to access the veterans’ activity log when using the default log-in	Provide clear instructions on the My HealtheVet log-in page to indicate that delegates should use a sign-in partner to view veterans’ account	
		Unable to log in to the delegation system	Enable proper log-in	
		System does not display username when logged in	Display the name of active account user	
**Domain: use**				
	**Clarity**			
		Secured email does not clearly distinguish between veteran and delegate senders	The author and subject line should distinguish secure emails from the veteran from those from the delegate	
	**Utility**			
		Providers do not have access to nonveteran delegate information	Include basic information on delegates (eg, sociodemographic data and relationship with veteran patient)	
		Delegates do not have the option to access veterans’ secure emails	System option for the delegate to obtain access to the veteran’s secure emails	
	**Functionality**			
		Notification is sent to either the veteran or delegate but not both	Provide a mechanism to notify both the veteran and delegate	
		New message notification is inaccurately displayed	Inbox notification should accurately display new messages	
		Save button on the My Profile page is not enabled	Enable the save button on the My Profile page	
		Secured email inaccurately displays the delegate’s name in the place of the veteran patient’s name when the delegate is also a veteran	Ensure accurate indication of the corresponding party (veteran, delegate, and provider)	

^a^VA: Department of Veterans Affairs.

^b^VDT: Veteran Delegation Tool.

#### Upgrade to My HealtheVet Premium Account

The process of advancing to a *Premium* account presents issues with accuracy and utility. For a delegate to access a veteran’s My HealtheVet account, a delegate must first create their own My HealtheVet account. Once a delegate’s My HealtheVet account is created, a *Premium* account upgrade is required to create VDT access. In theory, VDT will then grant the delegate access to the veteran’s My HealtheVet account. However, the language on the site indicates nonveterans and non-VA patients are ineligible for delegate registration, resulting in confusion owing to the lack of accuracy. To bypass this barrier to utility, delegates have to select a veteran or VA patient to successfully upgrade their My HealtheVet account to a *Premium* account.

#### VDT Registration

The participants identified several obstacles while attempting to register for VDT. They found inconsistencies with efficiency throughout registration. An instance of a lack of efficiency required veterans to provide authorization for their selected delegate to complete VDT registration. This process comprised multiple prompts for information and signatures from the veteran; the participants often reported feeling confused by the process. For example, to complete VDT registration, delegates must create 2 additional accounts, which requires navigating from My HealtheVet to an external secure log-on service; once completed, the registrant must return to the My HealtheVet platform. The process of accessing and navigating the activities across platforms created user confusion.

Additional obstacles were cited. First, regarding the usability factor of access, the participant registration process includes a limited invitation lifetime, a lack of standard operating procedure to register participants for the necessary sign-in partner, and an inability to complete registration without a *Premium* account. Learnability obstacles were exemplified when the participants found registration to be difficult owing to a lack of instruction or clear indication of task completion. In addition, the participants indicated not understanding the necessity of being directed to My HealtheVet to complete VDT registration as an obstacle to VDT affordance. They also identified unclear and misleading language as an obstacle to the intuitiveness of VDT.

**Figure 3:**
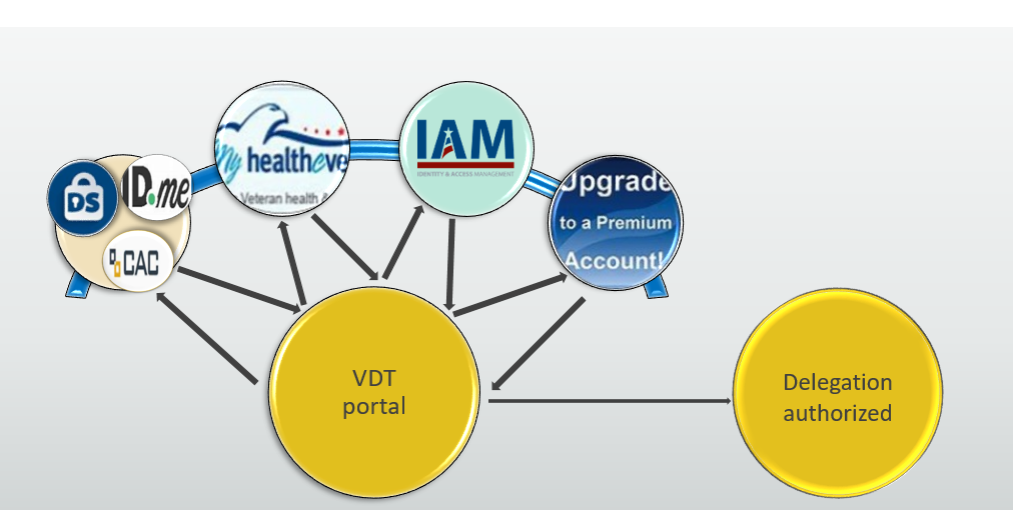
Journey mapping. VDT: Veteran Delegation Tool; ID.me: Online Identity Network Company; DS Logon: Department of Defense Self-service Logon; CAC: Common Access Card; I AM: Identity and Access Management Services.

Other errors related to functionality during registration included a mismatch of data when collecting personally identifiable information, a time lag between request for a delegate and sending invitation email, and an inability to process delegation authorization after veteran signature. The registration process is depicted in the journey mapping diagram (Figure 3).

#### Delegate Sign-in

For the protection of PHI, delegates must establish a secure sign-in using VA-approved sign-in partners. VDT offers multiple options to sign in but with minimal guidance. The options are relevant to specific populations, such as active-duty military or veterans; thus, the appropriate selection for civilian delegates lacks clarity and intuitiveness. Additional clarity concerns include unfamiliar language on the sign-in landing page, which reduces comprehension. Regarding functionality, user testing revealed several issues: the participants were not able to log on to VDT, delegate log-in did not navigate to the appropriate page, and user accounts did not display username.

#### VDT Use

VDT use is impacted by several HCD factors. Features of the VDT platform, such as secured email notification, were not accurately functioning at the time of the walkthrough. Another obstacle to functionality was that the providers were not always certain with whom they were communicating regarding veterans’ health information. The My HealtheVet system permits only one email address for within portal communication. Therefore, the identified email address from which the provider is contacted receives the provider's response regardless of who initially contacted the provider—the veteran or their delegate.

Access to MHV delegate information is limited for providers, thus challenging asynchronous and synchronous communication regarding the comanagement of veteran health care. Furthermore, delegates were not notified of new secure messages despite being identified as a delegate with those privileges. From the provider’s perspective, VDT lacked utility because the email correspondence did not clearly signify who the sender was, creating confusion and frustration.

## Discussion

### Overview

VDT, similar to other web-based and electronic platforms, warrants iterative HCD efforts to evolve into a useful and easy-to-use tool in preparation for national rollout targeted for care partners who are comanaging care with veterans. This project implemented an HCD process to examine users’ perceived benefits and facilitators of and barriers to using VDT and their practical experiences accessing and using the tool to inform recommendations for design enhancements and national implementation. VDT is a VHA priority to support the care partners of veterans in using VDT to comanage veterans’ health care needs.

### Principal Findings

Overall, the participants generally recognized the benefits and facilitators in promoting VDT use to include care partners in health care comanagement. Consistent with the literature, veterans and care partners recognized the need and benefit to be able to access web-based health information to comanage health care tasks [29]. The perceived benefits of accessing electronic health portals to manage health care have been firmly established in our previous work. VDT is an essential addition to the arsenal of tools needed to allow care partners and veterans to comanage veterans’ health care. Moreover, similar to our previous research [13-15], the confidentiality and protection of PHI was found to be highly valued by the targeted users when identifying determinant factors that promote or prevent the use of web-based health technology, such as VDT.

The participants perceived that VDT can enhance communication, especially during the COVID-19 pandemic to maintain access to remote care, when facing distancing restrictions [30,31]. These views are consistent with a recent study by Jackson et al [30] in which patients and care partners indicated that the use of a patient portal to read providers’ notes allows convenient and efficient ways to stay up to date with the patients’ treatment and health status. Current literature emphasizes that web-based assistance in managing health care is particularly important when care partners cannot attend clinic visits because they live apart from the patient or owing to COVID-19 distancing restrictions preventing care partners from attending clinical visits with veterans [30,32].

Aligned with recent literature, the users appreciated the bidirectional communication with authorized care partners [5]. In addition, the users valued the ability to clarify issues or make inquiries (eg, medications and appointments) without accompanying the veterans to clinic visits. Pandemic-related restrictions contextualized the imminent need for remote health care to maintain treatment and communication with patients and their care partners [32].

While the interviews provided compelling data about the perceived added value of VDT, understanding users’ practical experiences will ultimately determine the uptake and sustained use of VDT. User testing walkthroughs clearly indicated a series of issues related to access, navigation, and task completion, indicating that the users were often unable to complete the testing activities without experiencing adverse experiences. The complicated and clunky authentication and registration processes presented as a major barrier for the users. Complexity and lack of user ease throughout the registration process resulted in the tool being deemed too complicated for participants to successfully register for and use. Data clearly indicated a need for appropriate HCD enhancements to make VDT an easy-to-use, user-friendly tool for comanaging health care tasks.

### VDT Recommendations

On the basis of interview and user testing data and participant suggestions, we developed a list of recommendations and system enhancements, which are presented in Textbox 2, to improve VDT functionality to promote the uptake and sustained use of VDT. These recommendations were delivered to operational partners to inform VDT system enhancements to prepare for national implementation. The *design* and *deliver phases* are underway.

**List of recommendations and system enhancements to improve Veteran Delegation Tool (VDT) functionality.**

**Department of Veterans Affairs enterprise-wide access and interoperability:**

- Expand the capacity of the tool to integrate with other available tools and services
- Allow 3-way telehealth video appointments to include delegates as part of the care team
- Provide care partner access to a webpage to learn about eligibility for programs to facilitate health care management
- Interlink the Department of Veterans Affairs system with external providers to facilitate the sharing of information and connect internal and external providers
- Leverage the tool for care partners as a social support network to connect, exchange information, and find information relevant to their role
- Provide support via multiple means, including traditional conferencing calls and web-based appointments

**Minimize the barriers to register for and use VDT:**

- Simplify the registration and authorization processes; clarify the process with step-by-step guidance and provide a list of the documentation required to complete registration
- Create tutorial and onboarding information compatible with audience’s technical literacy
- Make users aware of their web browser’s compatibility before initiating the registration process
- Provide explanation of each sign-in partner option
- Grant automatic account access to designated delegates
- Provide notifications of completed tasks
- Display an option to search for a delegate’s name, similar to that of a patient search bar
- Clearly indicate the recipients of relevant messages

**VDT marketing and promotion:**

- Use promotional marketing strategies that explain the benefit of VDT for user groups
- Use diverse means of promotion, including passive promotion through brochures, flyers, posters, and electronic advertisements in waiting areas. Use active promotion, including word of mouth by peer veterans or through veteran groups and service organizations. Promote education and training resources to increase familiarity with VDT to help care partners fulfill their role as delegates, such as tutorials and step-by-step instructions
- Provide educational materials to the target audiences, including clinical team members, veteran patients, and their care partners. Materials developed for veterans and care partners should accommodate physical and literacy limitations
- Leverage the clinical team members in promotion; other recommendations included new patient orientation, direct staff support, and on-site demonstration where one can test the system and practice using its features

### Lessons Learned

The targeted users valued resources for comanaging care, and VDT was perceived as a potentially useful web-based health resource for users to remotely manage veterans’ health care. VDT enhancements are needed to improve accessibility and functionality for uptake and sustained use. Ongoing collaboration with stakeholder groups is necessary to identify or remediate functionality issues. Findings indicate that HCD is critical to improving the functionality of VDT implementation and sustainability. Improving the usability and promoting VDT will increase its future adoption, functionality, and effectiveness [33].

### Strengths and Limitations

The interdisciplinary collaborative HCD approach, putting veterans and care partners at the center of development, is this project’s primary strength. Working as an integrated team with diverse user groups permitted the use of collective efforts to identify and remediate VDT functionality issues. The integrated process also allowed an agile enhancement completion in response to the preliminary findings. This initial work lay the foundation for user testing to understand the usefulness, impact, and outcomes of VDT as perceived by intended users (ie, veterans, selected delegates, and clinical team members).

As with any quality improvement project, there are limitations that should be considered while acknowledging the practical nature of this work as an operationally partnered quality improvement project designed to integrate participatory mixed methods at a rapid iterative pace. First, the user testing analysis used a purposive sampling method, which may have led to a sampling bias. Second, the subsample size for user testing was limited to a total of 3 participants with 2 evaluation team *role players*. Previous work in the user testing field suggests using a rule of thumb such as 4 ± 1 to estimate the required number of users for simple projects [34]. It should be noted, due to the fatal user issues and experience during user testing, further user testing recruitment (n=3) was deemed unnecessary. Third, the limited functionality of VDT impacted the participants’ ability to fully use VDT. The limitations in VDT functionality restricted the initial interview phase to assessing the participants’ perceptions of potential benefits and barriers and gathering suggestions. A full functioning system would have allowed broader testing of the system with more concrete suggestions. Finally, the findings are limited to being generalized within the context of the VA health care system.

### Conclusions

The findings from this quality improvement project suggest that VDT can provide designated care partners formal access to veterans’ electronic health portal to allow trusted individuals to comanage a veteran’s health care needs while maintaining regulatory compliance. Delegation is an important tool to designate individuals comanaging veterans’ health care, but the tool must be accessible, functional, and easy to navigate and requires marketing promotion and education, especially for care partners as a new VA audience. Enhancements and iterative usability testing need to occur to establish a minimum viable product and develop performance measures to support the spread and sustainability of VDT. Marketing and education efforts tailored to the different user groups (eg, VA employees, veterans, and care partners) should be prioritized for the effective implementation and sustained use of VDT among the targeted user groups.

## Appendix

Multimedia Appendix 1 Veteran Delegation Tool interview guides.

## Abbreviations

HCD: human-centered design
PHI: personal health information
VA: Department of Veterans Affairs
VDT: Veteran Delegation Tool
VHA: Veterans Health Administration

## Data Availability

The data sets used and analyzed in this project are available from the corresponding author upon reasonable request.
