# Supplementary material for: User Testing of the Veteran Delegation Tool: Qualitative Inquiry
Source: J Med Internet Res. 2023 Feb 23;25:e40634. doi: 10.2196/40634 (PMC9999259; doi:10.2196/40634)
Supplement: Multimedia Appendix 1 [file jmir_v25i1e40634_app1.docx]

# **Veteran Interview guide**

# Participant ID____________ Interview date___________ Interview time________

# Relationship to On-line Healthcare Proxy_____________

Interviewer_____________ Rurality_______________

**Introduction**: Hello! Thank you for agreeing to talk with us today.

*Hello. My name is _________- I’m a health researcher with the Tampa VA, working with the Quality Improvement Team which seeks input from users and potential users of VA resources. In our current project we are reaching out to veterans to learn about their experiences and thoughts on VA electronic tools like* My Health*e*Vet, that intend to facilitate the effort to support Veterans.

*Your experiences and thoughts about the usefulness of such tools will inform the overall effort to promote services and ensure these meet the needs for Veterans like you.*

*We acknowledge that there are many levels of support that vary from individuals who help Veterans with appointments or that serves as an online healthcare proxy to receiving total support with daily activities and participation in the Caregiver Support program. We would like to better understand what this support means to you.*

*This interview will last approximately 45 minutes. We take notes during the interview and also like to record interviews to supplement our notes. Do we have your consent to record this conversation? Thank you! (interviewer to state: his/her name, Participant ID#, date, and time, Rurality).*

Today, we are going to talk about two existing tools Veterans use through the My Health***e***Vet system to manage their healthcare needs on-line. How familiar are you with the My Health***e***Vet system?

**If no…***The My HealtheVet portal has various tools that allow Veterans to create a free web-based Personal Health Record, access health education resources, and refill VA prescriptions.*

1. **Secure Messaging**-*This is* *an authorized electronic tool – a form of secure VA email - Veterans and their support system can use to communicate with VA Healthcare team members.*

***Using Secure Messaging***

1. How have you used Secure Messaging to date?

**Probe**: How comfortable do you feel sharing personal information through Secure Messaging?

**Probe**: How comfortable do you feel with the idea of someone you trust sharing your personal information through Secure Messaging?

1. What are some benefits to using Secure Messaging?

**Probe**: how might it affect your healthcare needs?

1. In light of the COVID-19 social distancing efforts, how has Secure Messaging influenced your care?
2. What are some barriers to using Secure Messaging?

**Probe**: What changes are needed for you to overcome these barriers?

1. What electronic devices do you use to manage your healthcare needs? Have you received any electronic devices from VA?

**Probe:** What tasks do you do on these devices?

**Probe:** How are these devices useful to you to manage healthcare related tasks?

**Probe:** What barriers do you experience, if any, when using these devices for your healthcare tasks?

1. *The next tool is the* ***Veteran Delegation Tool –*** *This tool lets you authorize a trusted individual like a spouse, adult child or friend to access your on-line medical health information such as medication refill and communicate via Secure Messaging with your healthcare providers. These trusted individuals are named ‘on-line healthcare proxy’. This tool is in final development stages. We hope to use your experience to make modifications to the current pilot tool.*

***Delegation – Usefulness***

1. Please describe your experience with the pilot Delegation tool.

**Probe**: What are the anticipated benefits of using this tool?

**Probe**: How will it change your communication with your healthcare team?

**Probe**: In light of the COVID-19 social distancing efforts, tell me how you foresee the

Delegation tool influencing your care.

1. What concerns do you have about using the Delegation tool?

**Probe:** What might help you overcome these concerns?

**Probe:** What would promote Veteran use of the Delegation tool?

1. What other tools that you currently use might work well with the Delegation to assist in co-managing your healthcare needs on-line?
2. If you choose to designate a trusted individual as your on-line healthcare proxy, how much of your healthcare information would you want them to have access to?
3. ***Evolving General Needs***

*We recognize that trusted individuals in your life may provide different levels supports over time. We would like to learn how they provide support. This includes a wide range of activities from keeping in touch with your healthcare providers to providing your physical care needs.*

What are some ways your (trusted individual, companion, spouse, etc.) supports your health care needs?

How has the type of support your (trusted individual, companion, spouse, etc.) provides changed over time?

How does your (trusted individual, companion, spouse, etc.) use electronic resources to assist you to meet our health care needs?

What can help you partner with your (trusted individual, companion, spouse, etc.) to manage your healthcare?

1. The VA offers support programs with a variety of services, how have you used these resources in the past? (**Prompts if needed**- Caregiver education, Self-care, Communication with healthcare providers, Navigating the VA system).

**Probe:** **If not** using VA support…. what are your reasons for not using the VA support/resources?

**Probe**: **If used**… how useful were these?

**Probe**: How have you learned about them?

**Probe**: What barriers did you encounter?

**Probe:** How might you overcome these barriers?

1. In light of the COVID-19 social distancing efforts, and the increased need for remote care, how has it impacted the way (trusted individual, companion, spouse, etc.) support your health care needs?

**Probe:** What additional resources would be useful to you and your trusted individual, companion, spouse, etc.).

1. ***Education***

How would you like to receive information on resources such as Delegation, Secure Messaging, and other tools?

What is your preferred way to learn about electronic tools such as the delegation tool?

1. **Closing**
2. Do you have any other comments or concerns you would like to share?

*We would like to reach out to you as additional tools and resources becomes available. Would you be interested in giving us additional feedback at that time?*

*That’s all for today. Thank you for your time.*

# **On-line Healthcare Proxy Interview guide**

# Participant ID____________ Interview date___________ Interview time________

# Relationship to Veteran_____________

Interviewer_____________ Rurality_______________

**Introduction**: Hello! Thank you for agreeing to talk with us today.

*Hello. My name is ___________- I’m a health researcher with the Tampa VA, working with the Quality Improvement Team which seeks input from users and potential users of VA resources. For our current project, we are reaching out to individuals like you who provide support to a Veteran in healthcare management or activities of daily living. This may include a role as an on-line healthcare proxy or participation in the Caregiver Support Program.*

*The purpose of the project is to learn about your experiences and thoughts on VA electronic tools, like My Health****e****Vet, that are part of the VA effort to promote services that support individuals like you. Your experiences and thoughts about the usefulness of such tools will inform the overall effort to promote services and ensure these efforts meet your needs.*

*This interview will last approximately 45 minutes. We take notes during the interview and also like to record interviews to supplement our notes. Do we have your consent to record this conversation? Thank you! (interviewer to state: his/her name, Participant ID#, date, and time, Rurality).*

*We acknowledge that there are many levels of support from just helping with appointment to providing total support. We would like to better understand what it means to you. Please use these categories to answer the next question. On a scale from 1-5, where 1 is “***SUPPORTIVE”**- Your healthcare support is *beneficial* to the Vet, but not necessary. Veteran is independent but appreciates the backing ***and 5 is*** *“***TOTAL SUPPORT”**- You are responsible for meeting the Veteran’s daily, health and mental health needs. You are unable to leave the Veteran unsupervised or unmonitored for more than a few hours.

1. How would you best describe the level of support that you typically provide for the Veteran over the past month? *[follow with reading the description to the number they give. If that makes them change the number up or down, read the new level. Do not need to read all of them].*

**Probe:** How does your level of support changes over time?

| **0**-**NONE**- You are not at all involved in the Veteran’s healthcare. The Vet prefers total privacy;  informal support not needed and unwanted. Veteran is completely independent.  **1**-**SUPPORTIVE**- Your healthcare support/co-management of the Veteran is *beneficial* to the Vet, but not necessary. Your contributions make better health outcomes possible. Veteran is independent but appreciates the backing.  **2**- **MINIMAL-** You might not have to do a lot, but your supplemental role contributes to the Veteran’s well-being. The Veteran is independent but is likely healthier and/or safer because of what you are willing to do.  **3**-**MODERATE**- You perform or assist in the performance of basic necessary activities of daily living for the Veteran. Without you, it would be challenging for the Veteran to adequately manage their own health or live independently. The Veteran might technically be independent but would likely suffer negative consequences without your help.  **4**-**MAXIMUM-** You are the reason the Veteran is able to live out in the community. You perform or directly support more than half of the activities necessary for daily living. The Vet is dependent on your support, otherwise would require institutional support.  **5**-**TOTAL**- You are responsible for meeting the Veteran’s ADL’s, IADL’s, and mental health needs. You are unable to leave the Veteran unsupervised or unmonitored for more than a few hours. |
| --- |

1. ***Evolving General Healthcare Proxy Needs***

*We recognize you may provide different supports over time to the Veteran in your life. We would like to learn how you provide that support.*

1. As your role evolves, what support do you need to meet Veteran healthcare needs?

**Probe**: What resources do you need that can help you partner with your Veteran to manage their healthcare?

**Probe**: How might an electronic or on-line platform assist you in accessing such support?

1. The VA offers support programs with a variety of services, how have you used these resources in the past? (**Prompts if needed**-Caregiver education, Self-care, Communication with healthcare providers, Navigating the VA system)

**Probe:** **If not** using VA support…. what are your reasons for not using the VA support/resources?

**Probe**: **If used**… how helpful were these?

**Probe**: How have you learned about them?

**Probe**: What barriers did you encounter?

**Probe:** How might you overcome these barriers?

1. In light of the COVID-19 social distancing efforts, and the increased need for remote care, what additional resources would be useful to you as a caregiver?

*Now, we are going to talk about two existing tools available for Veterans and individuals like you that can be found through the My Health****e****Vet system to manage healthcare needs on-line. How familiar are you with the My Health****e****Vet system?*

***If no…****The My HealtheVet portal has various tools that allow Veterans to create a free web-based Personal Health Record, access health education resources, and refill VA prescriptions.*

1. **Secure Messaging**-*This is* *an authorized electronic tool available on My HealtheVet– a form of secure VA email - Veterans and their support system use to communicate with VA Healthcare team members.*

***Using Secure Messaging***

1. What has been your experience using Secure Messaging to date?

**Probe**: How comfortable do you feel sharing information through Secure Messaging?

1. What are some benefits to using Secure Messaging?

**Probe**: How does Secure Messaging influence your ability to communicate Veteran healthcare needs?

1. In light of the COVID-19 social distancing efforts, how has Secure Messaging influenced Veteran care?
2. What are some barriers to using Secure Messaging?

**Probe**: What changes are needed for you to overcome these barriers?

1. What electronic devices do you use to manage your healthcare related tasks?

**Probe:** If he/she is an **official caregiver** ask “Have you received any electronic devices from the VA?

**Probe:** What tasks do you do on these devices?

**Probe:** How are these devices useful to manage healthcare related tasks?

**Probe:** What barriers do you experience, if any, when using these devices for your healthcare tasks?

1. *The next tool is the* ***Veteran Delegation Tool –*** *This tool lets Veterans authorize a trusted individual like yourself to access the Veteran medical health information on-line such as medication refill and communicate via Secure Messaging with Veteran healthcare providers. These trusted individuals are named ‘On-line Healthcare Proxy’. The Delegation tool is in final development stages. We hope to use your experience to make modifications to the current pilot tool.*

***Delegation - Usefulness***

1. Please describe your experience using the pilot Delegation tool.

**Probe**: What are the benefits of using this tool?

**Probe**: How has it change your communication with the VA healthcare team?

**Probe**: In light of the COVID-19 social distancing efforts, tell me how you foresee the Delegation tool influencing Veteran care.

1. What concerns do you have about using the Delegation tool?

**Probe:** What would promote the use of the Delegation tool?

1. As an On-line Healthcare Proxy, how much Veteran healthcare information would you want access to?
2. ***Education***

How would you like to receive information on resources such as Delegation, Secure Messaging, and other tools?

1. ***Closing***
2. Do you have any other comments or concerns you would like to share?

*We would like to reach out to you as additional tools and resources becomes available. Would you be interested in giving us additional feedback at that time?*

*That’s all for today. Thank you for your time.*
